# Bibliographical

**Published:** 1886-10

**Authors:** 


### BIBLIOGRAPHICAL.

### Dental Science, Questions and Answers.

DENTAL MATERIA MEDICA, DENTAL PHYSIOLOGY, DENTAL PATHOLOGY AND THERAPEUTICS.

By Luman C. Ingersoll, A. M., D. D. S., Dean of the Dental Department of the State University of Iowa.

This is a most excellent work, designed first of all for students, but may be read and re-read with great benefit by the practitioner as well.

Dr. Ingersoll has a happy faculty of presenting his thoughts in statements, brief, clear and understandable; qualities that are indispensable to the student and appreciated by others as well.

The purpose, as stated in the preface, is simply to bring conspicuously into notice the fundamental facts and principles which underlie the practice of dentistry. The style is that of question and answer. This is regarded by many as the most direct and explicit method of fixing attention upon the facts and principles to be learned, and as the most concise mode of formulating instruction.

As will be observed in the title page, the work contains three chapters. The first on Dental Materia Medica; the second, Dental Physiology; third, Dental Pathology and Therapeutics.

This is an excellent arrangement, and covers as much ground as any one teacher ought to attempt for a course of instruction. The catechetical method is calculated to stir new thoughts and suggestions in the mind of the reader; and that these may not be lost, every alternate leaf is a blank upon which the reader may make record of anything he may regard valuable for future use.

This work will prove a very valuable addition to our college class books. The book contains one hundred and thirty-six pages. It is neat in form and is well gotten up. It can be procured of the author, Keokuk, Iowa, and doubtless of any bookseller or Dental Depot. It ought to be in every dental office and in the hands of every dental student in the country.

### Index of Dental Periodical Literature.

This work which has been in preparation for several years, involving a good deal of careful labor, is just issued from the house of P. Blakiston, Son & Co., Philadelphia. In the preparation of the work the aim was to catalogue every paper, essay or article of any special importance or value in the entire English periodical literature of dentistry. A good degree of hope and expectation is entertained, that it may be of some service to those interested in the literature of our profession, and especially to students. It will enable them far more readily to gain a knowledge of what has been matter of record, than without such help. A list of the contributors to our periodicals, with the caption of a few of their more important productions is appended, which is not without interest, as showing who have been the workers in this direction.

The book contains two hundred and twelve pages. It may be obtained of booksellers and at the Dental Depots.

### The American System of Dentistry in Treatises by Various Authors.

Edited by Prof. W. F. Litch, M. D., D. D. S.

This work is to consist of three volumes of over one thousand pages each. The first volume is just at hand, too late for examination. Further notice will be made of it in the next number of the Register.

### Transactions of the Illinois State Dental Society, 1886.

This volume of 208 pages, filled with interesting and valuable matter, is at hand. This is the proceedings of one of the best, if not the best, State Society in the country. It should be read by every one interested in dental societies.

